# Adverse Events in Robotic Surgery: A Retrospective Study of 14 Years of FDA Data

**DOI:** 10.1371/journal.pone.0151470

**Published:** 2016-04-20

**Authors:** Homa Alemzadeh, Jaishankar Raman, Nancy Leveson, Zbigniew Kalbarczyk, Ravishankar K. Iyer

**Affiliations:** 1 Coordinated Science Laboratory, University of Illinois at Urbana-Champaign, Urbana, Illinois, United States of America; 2 Department of Surgery, Rush University Medical Center, Chicago, Illinois, United States of America; 3 Department of Aeronautics and Astronautics, Massachusetts Institute of Technology, Cambridge, Massachusetts, United States of America; Baylor College of Medicine, UNITED STATES

## Abstract

**Background:**

Use of robotic systems for minimally invasive surgery has rapidly increased during the last decade. Understanding the causes of adverse events and their impact on patients in robot-assisted surgery will help improve systems and operational practices to avoid incidents in the future.

**Methods:**

By developing an automated natural language processing tool, we performed a comprehensive analysis of the adverse events reported to the publicly available MAUDE database (maintained by the U.S. Food and Drug Administration) from 2000 to 2013. We determined the number of events reported per procedure and per surgical specialty, the most common types of device malfunctions and their impact on patients, and the potential causes for catastrophic events such as patient injuries and deaths.

**Results:**

During the study period, 144 deaths (1.4% of the 10,624 reports), 1,391 patient injuries (13.1%), and 8,061 device malfunctions (75.9%) were reported. The numbers of injury and death events per procedure have stayed relatively constant (mean = 83.4, 95% confidence interval (CI), 74.2–92.7 per 100,000 procedures) over the years. **Surgical specialties** for which robots are extensively used, such as gynecology and urology, had lower numbers of injuries, deaths, and conversions per procedure than more complex surgeries, such as cardiothoracic and head and neck (106.3 vs. 232.9 per 100,000 procedures, Risk Ratio = 2.2, 95% CI, 1.9–2.6). **Device and instrument malfunctions,** such as falling of burnt/broken pieces of instruments into the patient (14.7%), electrical arcing of instruments (10.5%), unintended operation of instruments (8.6%), system errors (5%), and video/imaging problems (2.6%), constituted a major part of the reports. **Device malfunctions impacted patients** in terms of injuries or procedure interruptions. In 1,104 (10.4%) of all the events, the procedure was interrupted to restart the system (3.1%), to convert the procedure to non-robotic techniques (7.3%), or to reschedule it (2.5%).

**Conclusions:**

Despite widespread adoption of robotic systems for minimally invasive surgery in the U.S., a non-negligible number of technical difficulties and complications are still being experienced during procedures. Adoption of advanced techniques in design and operation of robotic surgical systems and enhanced mechanisms for adverse event reporting may reduce these preventable incidents in the future.

## Introduction

During the last 14 years, over 1.75 million robotic procedures were performed in the United States across various surgical specialties [1]. Surgical robots enable conducting complex minimally invasive procedures with better visualization, increased precision, and enhanced dexterity compared to laparoscopy. Robotic devices provide 3-dimensional magnified views of the surgical field and translate the surgeon’s hand, wrist, and finger movements into precisely engineered movements of miniaturized surgical instruments inside patient’s body. The Intuitive Surgical’s da Vinci robot [2] is currently the only surgical robot approved by the U.S. Food and Drug Administration (FDA), for performing various types of procedures in urologic, gynecologic, general, cardiothoracic, and head and neck surgery. There are also other robotic systems designed for minimally invasive surgery in areas such as neurosurgery and orthopedic surgery (e.g. MAKO Surgical’s RIO Robotic Arm Interactive System for orthopedic surgery [3]) or for research in tele-operated robotic surgery (e.g. the da Vinci research kit [4] and the RAVEN II surgical robot [5][6]).

This study focuses on assessing the safety and effectiveness of robotic surgical systems used in minimally invasive surgery, by analyzing safety incidents experienced during robotic procedures. We retrieved all the nation-wide adverse event reports collected by the publicly available FDA MAUDE database [7] over the 14-year period of 2000–2013. We estimated the prevalence of incidents, including deaths, injuries, and device malfunctions over the years and across six major surgical specialties of gynecology, urology, general, colorectal, cardiothoracic, and head and neck surgery. We further characterized the potential causes for incidents and measured their impact on patients and on the progress of surgery.

There have been previous studies on safety and effectiveness of robotic surgery based on the experience of different surgical institutions as well as analyses of the FDA MAUDE data. However, an important question left unanswered is whether the evolution of the robotic systems with new technologies and safety features over the years has improved the safety of robotic systems and their effectiveness across different surgical specialties.

Our goal is to use the knowledge gained from the analysis of past incidents to provide insights on design of future robotic surgical systems that by taking advantage of advanced safety mechanisms, improved human machine interfaces, and enhanced safety training and operational practices can minimize the adverse impact on both the patients and surgical teams.

## Background and Related Work

Previous studies on the safety and effectiveness of minimally invasive robotic surgery focused on its comparison to non-robotic minimally invasive surgical methods (laparoscopy) and analysis of system failures experienced during procedures or the adverse events reported to the FDA MAUDE database. The FDA MAUDE data collected and analyzed in this manuscript cannot be used to determine the relative complication rates between robotic and non-robotic surgery. A summary of related work on such comparison is provided in S1 Table. This section presents an overview on the FDA MAUDE database and previous analyses of adverse events in robotic surgery.

### Failures of Robotic Surgical Systems

There have been several reports by individual surgical institutions on the various software-related, mechanical, and electrical failures experienced before or during robotic procedures [8]-[21]. S2 Table summarizes these reports by providing the number and types of procedures performed at each center as well as the failure rates and number of cases in which failures led to conversion or rescheduling of procedures. The rates of device malfunctions and failure-related conversions reported by these studies varied between 0.4–8.0% and 0.1–2.7%, with an average of 3% (95% confidence interval (CI), 1.9–4.2) and 0.9% (95% confidence interval (CI), 0.4–1.4), respectively.

### Adverse Events Reports from the FDA MAUDE Database

**Fig 1:**
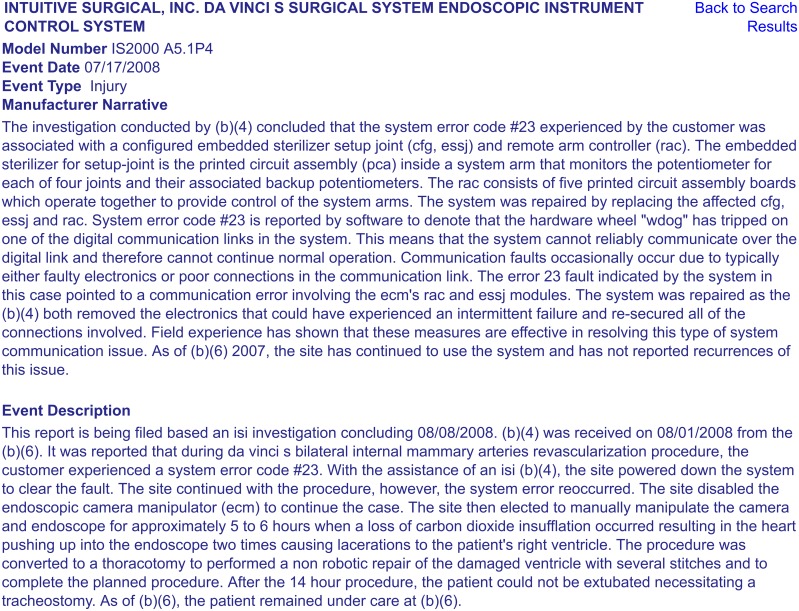
An example adverse event report from the publicly available FDA MAUDE database. This report is accessible through the online FDA MAUDE database at: http://www.accessdata.fda.gov/scripts/cdrh/cfdocs/cfMAUDE/Detail.cfm?MDRFOI__ID=2240665. Other MAUDE reports can also be accessed through searching the online database: http://www.accessdata.fda.gov/scripts/cdrh/cfdocs/cfMAUDE/TextSearch.cfm.

The Manufacturer and User Facility Device Experience (“MAUDE”) database is a publicly available collection of *suspected medical device-related* adverse event reports, submitted by mandatory (user facilities, manufacturers, and distributors) and voluntary (health care professionals, patients, and customers) reporters to the FDA [7]. Manufacturers and the FDA regularly monitor these reports to detect and correct device-related safety issues in a timely manner. Each adverse event report contains information such as *Device Name*; *Manufacturer Name*; *Event Type* (“Malfunction,” “Injury,” “Death,” or “Other”); *Event Date*; *Report Date*; and human-written *Event Description* and *Manufacturer Narrative* fields, which provide a short description of the incident, as well as any comments made or follow-up actions taken by the manufacturer to detect and address device problems [7]. Fig 1 shows an example MAUDE report on an adverse event occurred during a robotic cardiothoracic procedure.

The FDA MAUDE database, as a spontaneous reporting system, suffers from underreporting and inconsistencies [22][23][24]. However, it provides valuable insights on real incidents that occurred during the robotic procedures and impacted patient safety. The reported data on deaths, injuries, and device malfunctions provided by the MAUDE can be treated as a *sample set* to estimate the *lower bounds* on the prevalence of adverse events and identify examples of their major causes and patient impacts (see S1 File for more details on the problem of under-reporting).

Table 1 shows summary of related work on analysis of the FDA adverse event reports on robotic surgical systems. All these studies were performed by manual extraction and review of subsets of the MAUDE data. Almost half of the studies only focused on specific types of device failures (e.g., electro-cautery failures [25], electrosurgical injuries [26], and instrument failures [27]) and only considered the gynecology and urology specialties in their analysis. To the best of our knowledge, none of the previous studies considered the total volume of procedures performed in their analysis period and none of them assessed the risk of adverse events across different surgical specialties and their impact on the progress of surgery. This is in part due to the fact that adverse event descriptions in the FDA MAUDE database [7] are mainly composed of free-form natural language text, written by the manufacturers and healthcare professionals, and are often difficult to analyze without understanding semantics and the contextual factors involved in the events. Therefore, (i) manual analysis of incident reports requires significant amount of human effort, and (ii) often not all the contributing causes for incidents can be identified from the reports.

**Table 1:** Related work on analysis of the FDA adverse event reports on robotic surgical systems.

Study	No. Reports (Years)	System Under Study	Surgical Specialties	Major Results
Murphy et al. [25]	38 system failures, 78 adverse events (2006–2007)	da Vinci system	N/A	Most of these events were related to broken instrument tips or failures of electrocautery elements.
Andonian et al. [28]	189 (2000–2007)	ZEUS and da Vinci systems	N/A	Estimated failure rate of 0.38% for robotic-assisted laparoscopic surgeries.
Lucas et al. [29]	1,914 (2003 − 2009)	da Vinci system models dV and dVs	N/A	Both device malfunctions and open conversions were reduced by increased robotic experience and newer surgical systems. The number of patient injuries did not change and the number of deaths increased.
Fuller et al. [26]	605 (2001–2011)	da Vinci system	N/A	24 (3.9%) of reports were related to electrosurgical injuries (ESI), of which 37.5% resulted in surgical intervention.
Friedman et al. [27]	565 (2009–2010)	da Vinci Instruments	N/A	The majority of events were related to the instrument wrist or tip (285), 174 were related to cautery problems, 76 were shaft failures, and the rest were cable and control housing failures (36).
Gupta et al. [30]	741 (2009–2010)	da Vinci system	Urology, Gynecology	The events were related to the use of energy instruments (43.5%), surgical systems (19.3%), and the instruments (11.7%). The severity of events was correlated with the type of surgery and the type of device.
Manoucheri et al. [31]	50 injuries/deaths (2006–2012)	da Vinci system	Gynecology	The majority of injuries (65%) were not directly related to use of robot; 21% were related to operator error; and 14% were due to technical system failures.

## Methods

We developed a natural language processing tool that automatically retrieves all the reported events on robotic surgical systems from the FDA MAUDE database and extracts important safety information from the reports, including types of patient complications, surgical specialties and types of robotic procedures, most common types of system malfunctions, and the actions taken by the surgical teams to recover from failures. Our natural language parsing tool enables large-scale analysis of nation-wide incidents reported to the FDA over any timing period and, thus, facilitates more accurate estimation of the prevalence of incidents over the years and more effective evaluation of robotic surgical systems across different surgical specialties.

**Fig 2:**
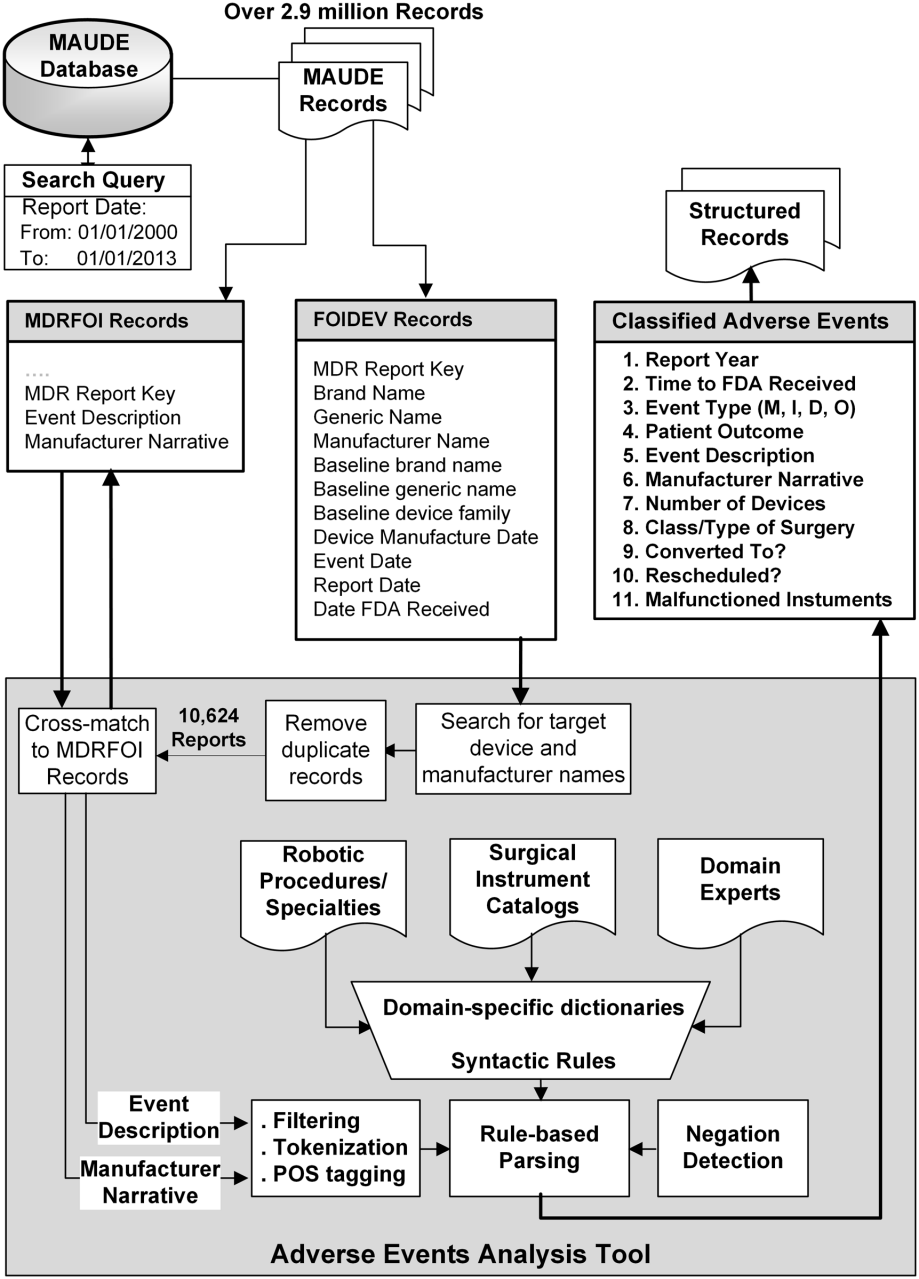
Data extraction and analysis flow from the FDA MAUDE database. The dictionaries of keywords for surgery types/specialties and surgical instruments were constructed based on the online information available on the da Vinci surgeries [32] and instruments catalog [33] from the manufacturer. The analysis tool was developed using open-source Python libraries for natural language processing, data analysis, and machine learning.

We extracted the reports related to the systems and instruments used in minimally invasive robotic surgery by searching for related keywords (e.g. device and manufacturer names) in the *Device Name* and *Manufacturer Name* fields of over 2.9 million MAUDE records posted between January 2000 and December 2013. That led us to an initial list of adverse event reports, from which we filtered out those with duplicate database keys (reporting the same adverse event for multiple devices). In addition to the structured information that was directly available from the reports, we extracted further information from the unstructured human-written descriptions of events by natural language parsing of the *Event Description* and *Manufacturer Narrative* fields (see Fig 1). As shown in Fig 2, our natural language processing tool combines domain knowledge with linguistic rules to interpret the semantics of the event descriptions. This was done by creating several domain-specific dictionaries (e.g., for patient complications, surgery types [32], surgical instruments [33], and malfunction types) as well as syntactic rules, parts-of-speech (POS) taggers, and negation detectors [34]. The results generated by each step of our automated analysis tool were manually reviewed for accuracy and validity.

More specifically, we extracted the following information from the reports:

- **Patient injury** (such as burns, cuts, or damage to organs) and death events that were reported under another *Event Type*, such as “Malfunction” or “Other”.
- **Surgical specialty and type of robotic procedure** during which the adverse events occurred.
- **Major types of device or instrument malfunctions** (e.g., falling of burnt/broken pieces of instruments into patients’ bodies or electrical arcing of instruments)
- **Adverse events that caused an interruption in the progress of surgery**, by leading the surgical team to troubleshoot technical problems (e.g., restarting the system), convert the procedure to non-robotic surgical approaches (e.g., laparoscopy or open surgery), or abort the procedure and reschedule it to a later time.

We compared the number of adverse events (in general) and injury/death events and procedure conversions (in particular) per 100,000 procedures across different surgical specialties. The rate of events was estimated by dividing the number of adverse events that occurred in each year (based on the *Event Date*) by the annual number of robotic procedures performed in the U.S. The total number of procedures per year was extracted from the device manufacturer’s reports [1][35] for 2004–2013 (see S1 Fig). The annual number of procedures per surgical specialty was available only for gynecology, urology, and general surgery after 2007. So we estimated a combined annual number of procedures for cardiothoracic and head and neck surgery by assuming that the majority of the remaining procedures (other than genecology, urology, and general) were related to these specialties, as, according to the manufacturer reports, they are the only other specialties for which the robot has been used [1]. Due to the possible underreporting of the adverse events in the FDA MAUDE database (the numerator) and the over-reporting of the annual number of robotic procedures in the manufacturing company investor presentations (the denominator), the estimated rates of adverse events per procedures are conservative and represent the *lower-bounds* on the prevalence of events.

We assumed that the rate of underreporting for injury and death events are low and are independent from the type of surgery, because the device manufacturers are required and monitored by the FDA to report serious injury and death events to the MAUDE database. However, due to possible changes in the reporting rates during the years, the total number of events per procedure in the whole study period (rather than per year) was compared across different surgical specialties. The 2-sided P values (< 0.05) and 95% confidence intervals were used to determine the statistical significance of the results. The cumulative number of malfunctions per procedure was used to evaluate the trends in malfunction rates over 2004–2013.

To characterize the major causes to which injury and death events were attributed, we performed a manual review of event descriptions for all the reports made before 2013. The cumulative number of malfunctions per procedure was used to evaluate the trends in malfunction rates over 2004–2013.

## Results

We identified a total of 10,624 events related to the robotic systems and instruments, reported over 2000–2013. About 98% of the events were reported by device manufacturers and distributors, and the rest (2%) were voluntary reports. In the same period, over 1,745,000 robotic procedures were performed in the U.S., so the estimated number of adverse events per procedure was less than 0.6% (95% confidence interval (CI), 0.6–0.62).

Data included 1,535 (14.4%) adverse events with significant negative patient impacts, including injuries (1,391 cases) and deaths (144 cases), and over 8,061 (75.9%) device malfunctions. For the rest of the events (1,028 cases), the *Event Type* information either was not available or was indicated as “Other.” We identified 160 adverse events (1.5%) that included some kind of patient injuries but were reported as a “Malfunction” or “Other.”

### Trends in Adverse Event Reports

**Fig 3:**
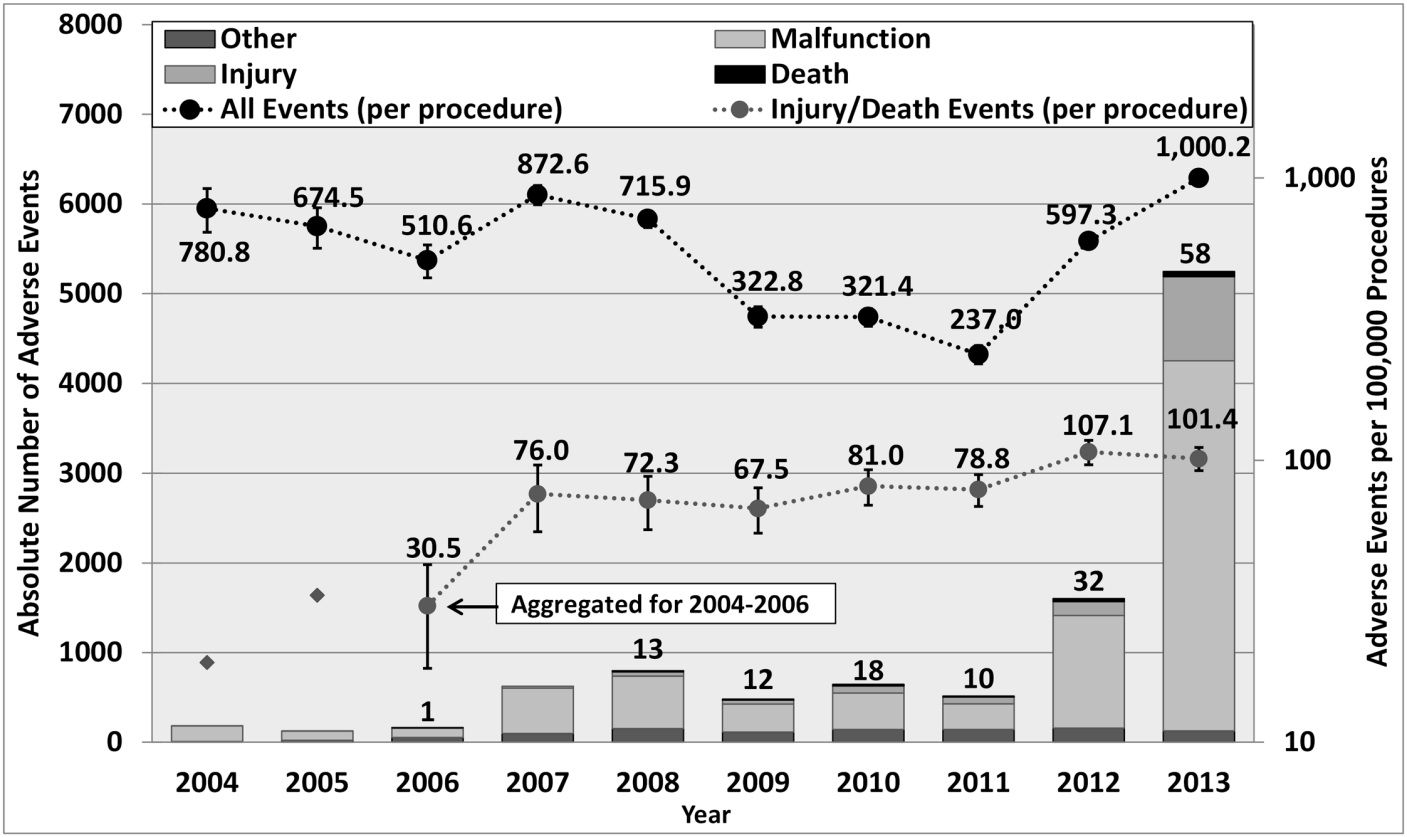
Annual Numbers of Adverse Event Reports and Rates of Events per Procedure. The left Y-axis corresponds to the bars showing the absolute numbers of adverse events (based on the years that reports were received by the FDA). The right Y-axis corresponds to the trend lines showing (in logarithmic scale) the annual number of adverse events per 100,000 procedures (based on the year the events occurred). Numbers on the bars indicate number of deaths reported per year. Error bars represent 95% confidence intervals for the proportion estimates. Because of the small number of injury and death events reported for 2004 and 2005, a combined rate was calculated for 2004–2006. Note that of all the events, 40 were reported as part of the articles or the legal disputes received by the manufacturer.

Fig 3 shows the overall trends in the annual numbers of reports and the estimated rates of events per 100,000 procedures over 2004–2013:

- The absolute number of reports made per year has significantly increased (about 32 times) since 2006, reaching 58 deaths, 938 patient injuries, and 4,124 malfunctions in 2013. The number of robotic procedures performed per year has increased 10-fold in the same period [1][35].
- While the annual average number of adverse events was about 550 per 100,000 procedures (95% confidence interval (CI), 410–700) between 2004 and 2011, in 2013 it peaked at about 1,000 events per 100,000 procedures (1 event reported in every 100 procedures).
- The numbers of injury and death events per procedure have stayed relatively constant since 2007 (mean = 83.4 per 100,000 procedures, 95% CI, 74.2–92.7).

### Adverse Events across Different Surgical Specialties

Table 2 shows the numbers of adverse events reported in different surgical specialties and their impact on patients (injuries or deaths) and progress of surgery (procedure conversion or rescheduling). The last row shows examples of the most common types of procedures reported in each specialty.

**Table 2:** Adverse events in different surgical specialties: Deaths, injuries, malfunctions, procedure conversion or rescheduling, common types of surgery.

	No. (%) [95% Confidence Interval]						
	Gynecology	Urology	Cardiothoracic	Head & Neck	Colorectal	General	N/A
	**3,194**	**1,565**	393	71	301	197	4,903
**Overall** ^a^	**(30.1)**	**(14.7)**	(3.7)	(0.7)	(2.8)	(1.9)	(46.2)
	**[29.2–31.0]**	**[14.0–15.4]**	[3.3–4.1]	[0.5–0.9]	[2.5–3.1]	[1.6–2.2]	[45.3–47.1]
**Event Type** ^b^							
Death	46	30	25	**14**	11	11	7
	(1.4)	(1.9)	(6.4)	**(19.7)**	(3.7)	(5.6)	(0.1)
	[1.0–1.8]	[1.2–2.6]	[4.0–8.8]	**[10.4–29.0]**	[1.6–5.8]	[2.4–8.8]	[0.0–0.2]
Injury	**818**	272	64	14	58	**56**	109
	**(25.6)**	(17.4)	(16.3)	(19.7)	(19.3)	**(28.4)**	(2.2)
	**[24.1–27.1]**	[15.5–19.3]	[12.6–20.0]	[10.4–29.0]	[14.8–23.8]	**[22.1–34.7]**	[1.8–2.6]
Malfunction	2,103	902	226	35	209	110	4,476
	(65.8)	(57.6)	(57.5)	(49.3)	(69.4)	(57.8)	(91.3)
	[64.2–67.4]	[55.2–60.0]	[52.6–62.4]	[37.7–60.9]	[64.2–74.6]	[48.9–62.7]	[90.5–92.1]
Other	227	361	78	8	23	20	311
	(7.1)	(23.1)	(19.8)	(11.3)	(7.6)	(10.2)	(6.3)
	[6.2–8.0]	[21.0–25.2]	[15.9–23.8]	[3.9–18.7]	[4.6–10.6]	[6.0–14.4]	[5.6–7.0]
	236	**212**	**66**	6	29	14	217
**Conversion**	(7.4)	**(13.5)**	**(16.8)**	(8.5)	(9.6)	(7.1)	(4.4)
	[6.5–8.3]	**[11.8–15.2]**	**[13.1–20.5]**	[2.0–15.0]	[6.3–12.9]	[3.5–10.7]	[3.8–5.0]
	26	**148**	**11**	1	1	**6**	77
**Rescheduling**	(0.8)	**(9.5)**	**(2.8)**	(1.4)	(0.3)	**(3.0)**	(1.6)
	[0.5–1.1]	**[8.1–10.9]**	**[1.2–4.4]**	[0–4.1]	[0–1.0]	**[0.6–5.4]**	[1.3–1.9]
**Common Surgery Types**	Hysterectomy (2,331)	Prostatectomy (1,291)	Thoracic (110)	Thyroidectomy (19)	Cholecyst-ectomy (118)	Hernia repair (37)	
	Myomectomy (328)	Nephrectomy (138)	Lobectomy (67)	Tongue base resection (19)	Colectomy (61)	Nissen fundoplication (34)	
	Sacrocolpopexy (170)	Pyeloplasty (31)	Mitral valve repair (54)	Transoral robotic (18)	Low anterior resection (44)	Gastric bypass (28)	
	Oophorectomy (120)	Cystectomy (48)	Coronary artery bypass (23)		Colon resection (25)	Gastrectomy (15)	

^a^ Percentages are over all the adverse event reports (n = 10,624).

^b^ Percentages are over the total adverse events reported for a surgical specialty.

- The majority of reports were related to gynecology (30.1%), urology (14.7%), and cardiothoracic (3.7%) surgeries, such as hysterectomy (2,331), prostatectomy (1,291), and thoracic (110) procedures, respectively. The higher percentage of adverse events in gynecologic and urologic surgeries could be explained by the higher number of these procedures performed (86% of all the robotic procedures performed in the U.S.) compared to other surgical specialties (less than 14.2% of all procedures) [1].
- Cardiothoracic and head and neck surgeries involved a higher number of deaths per adverse event report (6.4% and 19.7%) than gynecology and urology (1.4 and 1.9%).
- The highest number of procedure conversions per adverse event was for cardiothoracic (16.8%) and urology (13.5%), and the highest rates of procedure rescheduling were for urology (9.5%), general (3.0%), and cardiothoracic (2.8%) surgeries.

Of all the reports, only 5,721 (53.8%) indicated the class and type of surgery involved. However, the majority of reports with missing information on the type of surgery were related to device malfunctions and “Other” events (97.6%). In order to compare the rate of adverse events across different specialties, we focused only on reports related to injuries, deaths, and procedure conversions. For the majority of these events (92.2% of injury reports, 95.1% of deaths, and 72.2% of procedure conversions), the surgery type information was available and the rest (with ‘N/A’ surgical specialty) were removed from our analysis. In order to estimate the rate of events per procedure, we regrouped the events into four major categories of “Gynecology,” “Urology,” “General,” and “Cardiothoracic and Head and Neck,” according to the manufacturer’s reports [1][35]. The “General” category includes both colorectal and general specialties.

As shown in Table 3, for cardiothoracic and head and neck surgery, the rates of injuries, deaths, and procedure conversions have been significantly higher than other specialties. During 2007–2013, the estimated rate of deaths have been 52.2 per 100,000 procedures for cardiothoracic and head and neck specialties vs. 5.7 in gynecology, urology, and general surgeries (RR = 9.23, 95% CI, 6.35–13.40, P < 0.0001). Also, the rate of injuries and procedure conversions in these specialties have been 91.0 and 89.7 per 100,000 procedures vs. 71.5 (RR = 1.27, 95% CI, 0.99–1.63, P < 0.052) and 29.2 (RR = 3.07, 95% CI, 2.38–3.97, P < 0.0001) in the other surgical categories.

**Table 3:** Comparsion of adverse events rates in different surgical specialities (2007–2013).

	No. (rate per 100,000 procedures) ^a^ [95% CI]					
	Gynecology, Urology, General		Cardiothoracic, Head and Neck, Other		Cardiothoracic and Head and Neck vs. Gynecology, Urology, and General	
Total Procedures	1,661,891		74,709		**Relative Risk (95% Cl)**^b^	
Total Adverse Events	5,209		447		***P* Value**	
Event Type						
Death	94	(5.7)	39	(52.2)	9.23 (6.35–13.40)	< 0.0001
Injury	1188	(71.5)	68	(91.0)	1.27 (0.99–1.63)	< 0.052
Conversion	485	(29.2)	67	(89.7)	3.07 (2.38–3.97)	< 0.0001
Rescheduling	180	(10.8)	12	(16.1)	1.48 (0.83–2.66)	< 0.19 ^c^

^a^ Percentages are over total number of procedures in each column.

^b^ Assuming that the level of underreporting across different surgical specialties is similar.

^c^ Not statistically significant because of the small number of samples (12) in the cardiothoracic and head and neck surgery.

### Device and Instrument Malfunctions

We identified five major categories of device and instrument malfunctions that impacted the patients, either by causing injuries and complications or by interrupting the progress of surgery and/or prolonging procedure times. Table 4 shows the numbers of events in each category, the event types as indicated by reporters (including “Malfunction” (M), “Injury” (IN), “Death” (D), and “Other” (O)), and the actions taken by the surgical team to resolve the problems. The malfunction categories and actions taken by the surgical teams are not mutually exclusive, and in many cases two or three different malfunctions or two actions were reported in a single event. S2 and S3 Figs. use Venn diagrams to depict the intersections among different malfunction categories and actions taken by the surgical team.

**Table 4:** Major categories of malfunctions. (Note that the malfunction and surgical team action categories are not mutually exclusive, i.e., in many cases more than one malfunction or action were reported in a single event.)

Malfunction Category		No. of Reports					Surgical Team Actions (% of malfunction category)		
	Description	Total	Event Type				System Reset	ProcedureConverted	Procedure Rescheduled
		(% of all)	M	IN	D	O			
**System**	- System error codes and faults	536					**231**	**330**	**133**
**Errors**	- System transferred into a recoverable or non-recoverable safety state	(5.0%)	44	23	1	468	**(43.1%)**	**(61.6%)**	**(24.8%)**
**Video/**	- Loss of video	275	21	18	0	236	**53**	**145**	**94**
**Imaging Problems**	- Display of blurry images at surgeon’s console or assistant’s touchscreen	(2.6%)					**(19.3%)**	**(52.7%)**	**(34.2%)**
**Broken**	- Burnt/broken parts and components								
**Pieces**	- Fell into surgical field or body cavity	**1,557**	1,396	119	1	41	3	38	5
**Falling Into Patients**	- Required additional procedure time to be found/removed from the patient	**(14.7%)**					(0.2%)	(2.4%)	(0.3%)
**Broken Tip**	- Tears, burns, splits, holes on tip cover	**1,111**	900	193	0	18	2	18	0
**Covers or Elec. Arcing**	- Electrical arcing, sparking, charring	**(10.5%)**					(0.2%)	(1.6%)	(0.0%)
**Unintended Instrument**	- Unintended or unstoppable movements started without the surgeon’s command	**1,078**	919	52	2	105	31	93	21
**Operation**	- Instruments not recognized by system	**(10.1%)**					(2.9%)	(8.6%)	(1.9%)
	- Instruments not working, open/closed								
**Other**	- Cable, wire, tube, or instrument damages and breakages	5,092	4,962	55	1	74	20	62	13
	- Issues with electrosurgical units, power supplies/cords, patient-side manipulators, etc.	(47.9%)					(0.4%)	(1.2%)	(0.3%)
	- Other events reported as “Malfunction								
**Total**	- All malfunctions	9,377	8,061	443	5	868	305	630	246
**(% of all)**		(88.3%)					(3.3%)	(6.7%)	(2.6%)
	**All Adverse Events**	10,624	**8,061**	**1,391**	**144**	**1,028**	**334**	**780**	**270**
		(100%)					**(3.1%)**	**(7.3%)**	**(2.5%)**

- **System errors and video/imaging problems** contributed to 787 (7.4%) of the adverse events and were the major contributors to procedure interruptions, including system resets (274 cases, 82% of all system resets), conversion of the procedures to a non-robotic approach (462 cases, 59.2% of all conversions), and aborting/rescheduling of the procedures (221 cases, 81.8% of all cases). System errors are raised by the existing safety mechanisms of the robot upon detection of device problems that cannot be autonomously recovered from and either require manual system reset (recoverable system errors) or stopping the robotic procedure (non-recoverable system errors). S3 Table lists the descriptions and frequencies of the most common system error codes that we extracted by natural language parsing of the adverse event descriptions in the reports.
- **Falling of the broken/burnt pieces into the patient’s body** constituted about 1,557 (14.7%) of the adverse events. In almost all these cases, the procedure was interrupted, and the surgical team spent some time searching for the missing pieces and retrieving them from the patient (in 119 cases, a patient injury, and in one case a death, was reported).
- **Electrical arcing, sparking, or charring of instruments** and burns or holes developed in the tip cover accessories constituted 1,111 reports (10.5% of the events), leading to nearly 193 injuries, such as burning of tissues.
- **Unintended operation of instruments**, such as uncontrolled movements and spontaneous powering on/off, happened in 1,078 of the adverse events (10.1%), including 52 injuries and 2 deaths.

The *Other* category in Table 4 represents the malfunctions that could not be classified in any of the classes, including cable and instrument breakages that did not lead to other types of malfunctions, electrosurgical unit and power supply problems, and patient-side manipulator issues.

In total, 9,377 reports were about technical problems, including 1,104 cases (10.4% of all the adverse events) in which the procedure was interrupted and additional time was spent on troubleshooting the errors, resetting the system, and/or converting the procedure to a traditional technique, or rescheduling the procedure to a later time. In 1,019 of cases (10.9% of all the malfunctions), the device or instrument malfunction was detected prior to start of the procedure, of which in 20 cases the procedure was rescheduled to a later time and in 2 cases it was converted to a non-robotic approach.

**Fig 4:**
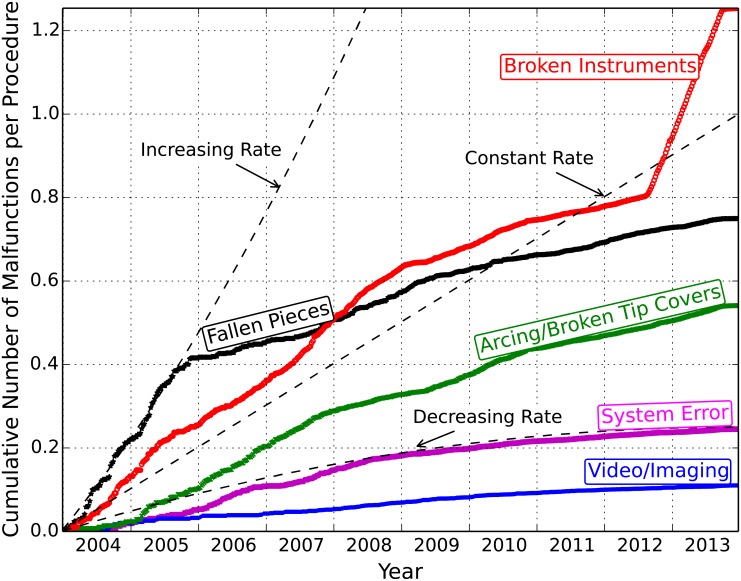
Cumulative rates of malfunctions per procedure. The rates of malfunctions per procedure were obtained for each week (see S1 Fig for more details on the estimation of the number of procedures).

Fig 4 shows the cumulative rates of malfunctions per procedure over 2004–2013. Overall, the malfunction rates decreased after 2006, but the rate of cases with arcing instruments and broken instruments followed a relatively constant trend. The sudden increase in the rate of broken instruments after the middle of 2012 could be related to changes made to the adverse event reporting practices by the manufacturer in 2012 (mostly related to instrument cable breaks) [36], as well as increased reporting of adverse events after concerns about the safety of robotic surgery were raised by the FDA [37][38] and public media in early 2013 [39][40][41].

### Injury and Death Causes

A manual review of a sample set of injury and death reports (from 2000–2012) allowed us to classify the causes indicated by reporters into three main categories: inherent risks associated with surgery, technical issues with the robot, and mistakes made by the surgical team. For the majority of death events, little or no information was provided in the reports. About 50% of the death events were indicated by the reporters to be related to inherent risks or complications of surgery, 11.6% due to patient’s history or health state, and 7% were attributed to surgeon/staff mistakes (e.g., incorrect instrument change or accidental cuts of artery). Of all the reported deaths in different classes of surgery, at least 75.3% (64 of 86) happened after the procedure (mainly due to patient history, infection/sepsis, or uncontrollable and heavy bleeding) and 17.4% (15 of 86) happened during the procedure. Of the deaths that occurred during the procedures, five were due to inadvertent cuts or punctures of organs and the others were related to complications such as uncontrolled bleeding, pulmonary embolism, and cardiac arrest.

As Table 5 shows, about 62% of the injury events involved device malfunctions and the rest were related to operator errors (7.1%), improper positioning of patient or port incisions (6.3%), inherent risks of surgery (3.9%), or problems with grounding the equipment (1.5%). S4 Table lists example reports on device malfunctions that impacted patients during cardiothoracic procedures.

**Table 5:** Summary of death and injury reports (2000–2012).

	**Death Reports (Total = 86)**	
	**Example Causes**	**Number of Reports (%)**
	Surgeon/staff mistake	6 (7.0%)
	Patient’s history	10 (11.6%)
	Inherent risks and complications	43 (50.0%)
	N/A	27 (31.4%)
**During Procedure**	Punctures, bleeding, pulmonary embolism, cardiac arrest	15 (17.4%)
**After Procedure**	Infection/sepsis, heavy bleeding	64 (75.3%)
**Injury Reports (Total = 410)**		
**Example Causes**		**Number of Reports (%)**
Device malfunctions		254 (62.0%)
Surgeon/staff mistake		29 (7.1%)
Improper positioning of the patient led to post-operation complications such as nerve damage		17 (4.1%)
Inherent risks of surgery and patient history		16 (3.9%)
Burning of tissues near port incisions		9 (2.2%)
Possible passing of the electrosurgical unit currents through instruments to the patient body		6 (1.5%)
Surgeon felt shocking at the surgeon-side console		2 (0.5%)
N/A		77 (18.8%)

A more in-depth review of several injury and death reports showed that adverse events are often not due to a single root cause (e.g., a given component failure or a human error), but a combination of multiple causal factors and underlying conditions that lead to safety hazards and incidents. For example, the report shown in Fig 1 (MAUDE report no. 2240665), describes an event where an electronic component failure, a non-recoverable system error, inadequate training and troubleshooting procedures for dealing with technical problems (non-recoverable system errors), and possibly ineffective decisions made by human operators (surgeon deciding to manually operate the endoscopic camera instead of converting the procedure) each played a role in a very long procedure time, loss of carbon dioxide insufflation, and consequently, a patient injury. The following are the common flawed operational practices used by the surgical team that contributed to catastrophic events during surgery:

- Inadequate experience with handling emergency situations
- Lack of training with specific system features
- Inadequate troubleshooting of technical problems
- Inadequate system/instrument checks before procedure
- Incorrect port placements
- Incorrect electro-cautery settings
- Incorrect cable connections
- Inadequate manipulation of robot master controls
- Inadequate coordination between hand & foot movements
- Incorrect manipulation or exchange of instruments

A more comprehensive analysis of multi-dimensional causes of incidents is the topic of the future research.

## Discussion

Our analysis shows an increasing number of adverse events related to the robotic surgical systems being reported. As cautioned by the FDA [7][37], the number of MAUDE reports may not be used to evaluate the changes in rates of events over time, because the increased reporting of events may be due to different factors, e.g., the increasing use of surgical systems [1], changes in the manufacturers’ reporting practices [36], and/or better awareness and increased publicity resulting from product recalls, media coverage, and litigation [37]. Therefore, we measured the prevalence of adverse events in each year by estimating the number of events reported *per procedure*. We found that despite a relatively high number of reports, the vast majority of procedures were successful and did not involve any problems and the number of injuries/death events per procedure has stayed relatively constant since 2007. However, total number of malfunctions reported per procedure (0.46%, 95% confidence interval (CI), 0.45–0.47%) was about 6 times lower than the average number of malfunctions per procedure (3%, 95% confidence interval (CI), 1.9–4.2) published by different surgical institutions (see S2 Table). Also the total number of injuries and deaths reported per procedure (0.08%, 95% confidence interval (CI), 0.08–0.09%) was about the same as the predicted complication rates for robotic surgery [42] but an order of magnitude less than the lowest rate of complications reported for robotic surgery in previous studies (2% [43]) (see S1 Table). This further confirms the uncertainty in the rates of events due to possible under-reporting in MAUDE data and possible changes in reporting practices.

Our analysis shows that estimated number of events per procedure in complex surgical areas, such as cardiothoracic and head and neck surgery were significantly higher than gynecology, urology, and general surgeries. Although not all the reported injuries and deaths were due to device problems, and the procedure conversions, of themselves, cannot be considered adverse events [44][45], the estimated numbers of injury/death events and conversions per procedure can be used as a metric to measure the difficulty experienced in different surgical specialties. The best that we can tell from the available data is that the higher number of injury, death, and conversion *per adverse event*, in cardiothoracic and head and neck surgeries, could be indirectly explained by the higher complexity of the procedures, less frequent use of robotic devices, and less robotic expertise in these fields. Although the use of robotic technology has rapidly grown in urology and gynecology for prostatectomy and hysterectomy, it has been slow to percolate into more complex areas of cardiothoracic and head and neck surgery. Between 2007 and 2013, over 1.4 million (86%) robotic procedures in gynecology and urology were performed in the U.S., while the number of procedures in other surgical specialties altogether was less than 250,000 (14.2%) [1]. The limitations of the robotic user interface [46], long procedure times [47], steep learning curve [48][49], and higher costs for purchase and maintenance of robotic systems and instruments [50] are some factors that may have contributed to the lower utilization of the robotic approach in more complex surgical procedures. For example, only a select type of robotic cardiac procedures are reported to have been successfully performed using the robots, such as mitral valve repair and internal mammary artery harvest [51][52][53]. The experiences of highly competent robotic teams that performed multi-vessel coronary artery bypass grafting (CABG) showed that the robotic approach may be associated with higher mortality and morbidity rates compared to open surgery [54]. In head and surgery, there may be the problem of close anatomical proximity to many vascular and neurological structures that may increase procedural complexity. Also, bleeding when it occurs may be difficult to control because of increased vascularity.

In practice, the use of the robotic platform involves the interface of a sophisticated machine with surgical teams, in an area of patient care that is safety-critical. From a technology perspective, employing substantially improved safety practices and controls in the design, operation, and validation of robotic surgical systems could prevent some of the reported events. Some examples include:

- Improved human-machine interfaces and surgical simulators that train surgical teams for handling technical problems [55][56] and assess their actions in real-time during the surgery.
- Providing real-time feedback to the surgeon on the safe surgical paths that can be taken or safety barriers that prevent the robotic tools to enter to certain portions in the surgical workspace [57], based on the patient-specific anatomical models, as well as surgeon-specific modeling and monitoring of robotic surgical motions [58], to minimize the risk of approaching dangerous limits and inadvertent patient injuries.
- New safety engines for monitoring of procedures (including surgeon, patient, and device status) and providing comprehensive feedback to surgical team on upcoming events and troubleshooting procedures to prevent long procedure interruptions.
- Improved mechanisms for logging and reporting of incidents experienced during procedures to enable more accurate validation of safety and effectiveness of surgical systems.

### Limitations

The results of our study come with the caveats that: (i) inherent risks exist in all surgical procedures (more so in complex procedures), (ii) the non-device-related adverse events (e.g., caused by human errors) are less likely to be reported to the MAUDE database, and (iii) the MAUDE database suffers from underreporting and inconsistencies. Thus, the estimated number of adverse events per procedure are likely to be lower than the actual numbers in robotic surgery. Further, the lack of detailed information in the reports makes it difficult to determine the exact causes and circumstances underlying the events. Therefore, the sensitivity of adverse event trends to changes in reporting mechanisms, surgical team expertise, and inherent risks of surgery could not be assessed based on this data.

## Conclusions

While the robotic surgical systems have been successfully adopted in many different specialties, this study demonstrates several important findings: (i) the overall numbers of injury and death events per procedure have stayed relatively constant over the years, (ii) the probability of events in complex surgical specialties of cardiothoracic and head and neck surgery has been higher than other specialties, (iii) device and instrument malfunctions have affected thousands of patients and surgical teams by causing complications and prolonged procedure times.

As the surgical systems continue to evolve with new technologies, uniform standards for surgical team training, advanced human machine interfaces, improved accident investigation and reporting mechanisms, and safety-based design techniques should be developed to reduce incident rates in the future.

## Supporting Information

S1 Fig Estimated numbers of procedures performed during 2004–2013. The annual numbers of procedures performed in the U.S. for 2010–2013 were extracted from the annual reports of the manufacturer. For 2004–2009, we estimated the numbers of procedures by measuring the graphs in the robot manufacturer’s investor presentations. Whenever the estimated numbers from two different sources did not match or the data were available only for the total worldwide procedures, we chose the maximum number of procedures for that year in order to achieve a lower bound on the likelihood of events.
We estimated the number of procedures per week from annual number of procedures by fitting a 4-degree polynomial curve (R^2^ = 0.999) to the bar graph of annual procedures and calculating the area under the fitted curve for every week.
(EPS)

S2 Fig Intersections among different malfunction categories. A total of 3,067 adverse event reports were not classified by MedSafe in any of the malfunction categories.
(TIFF)

S3 Fig Intersections among system resets, converted, and rescheduled cases. For 9,520 of adverse events, no system reset, conversion, or rescheduling were reported.
(TIFF)

S1 File Underreporting. (PDF)

S1 Table Robotic versus non-robotic surgical methods. (PDF)

S2 Table Summary of related work on failures of robotic surgical systems. (PDF)

S3 Table Most frequent system error codes. (PDF)

S4 Table Example malfunctions and their patient impact during cardiothoracic procedures. (PDF)
