# Supplementary figures and images for: Adverse Events in Robotic Surgery: A Retrospective Study of 14 Years of FDA Data
Source: PLoS One. 2016 Apr 20;11(4):e0151470. doi: 10.1371/journal.pone.0151470 (PMC4838256; doi:10.1371/journal.pone.0151470)

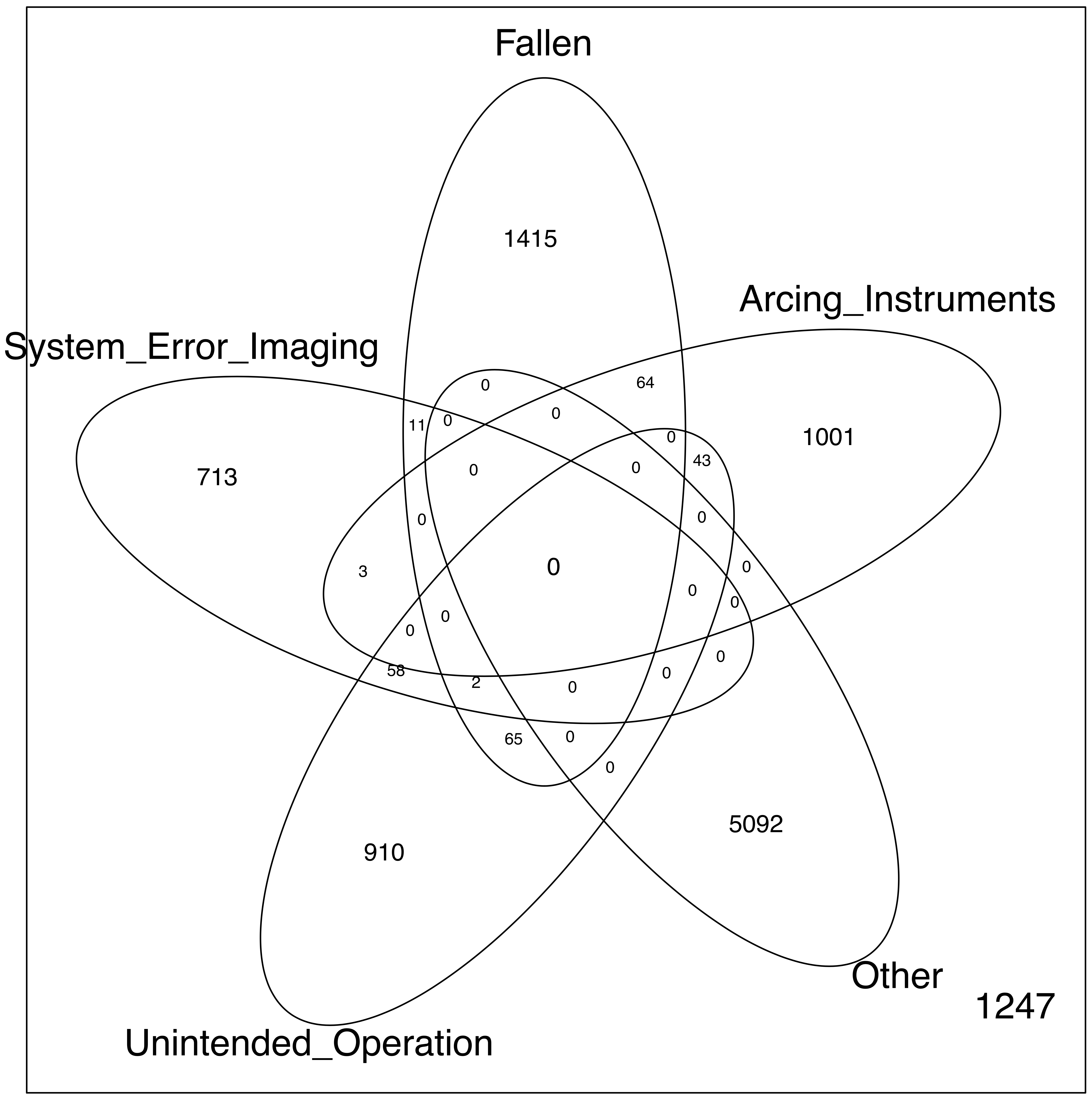

Supplement: S2 Fig — A total of 3,067 adverse event reports were not classified by MedSafe in any of the malfunction categories. (TIFF) [file pone.0151470.s002.tiff]

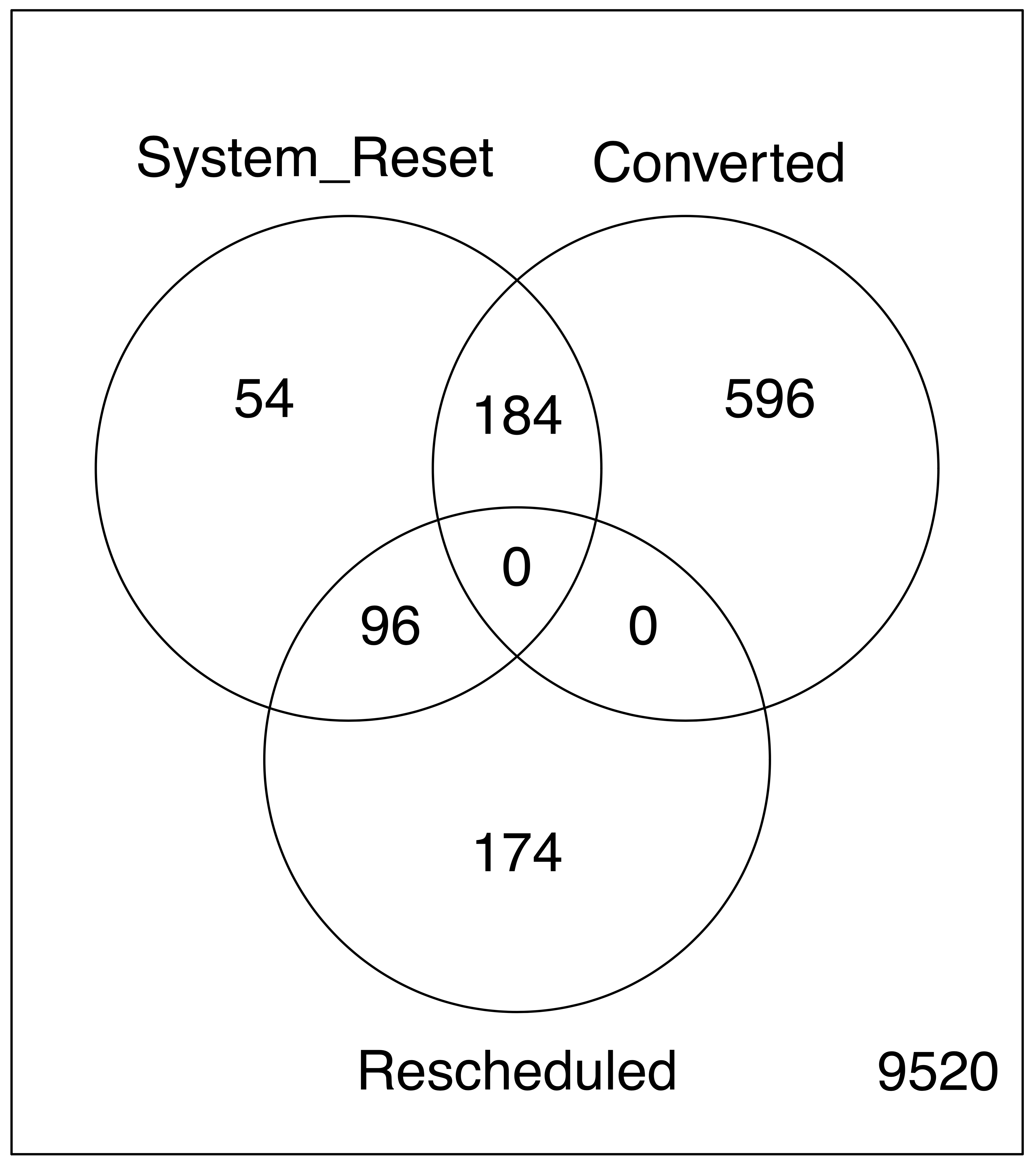

Supplement: S3 Fig — For 9,520 of adverse events, no system reset, conversion, or rescheduling were reported. (TIFF) [file pone.0151470.s003.tiff]
