# Supplementary material for: Adverse Events in Robotic Surgery: A Retrospective Study of 14 Years of FDA Data
Source: PLoS One. 2016 Apr 20;11(4):e0151470. doi: 10.1371/journal.pone.0151470 (PMC4838256; doi:10.1371/journal.pone.0151470)
Supplement: S1 File — (PDF) [file pone.0151470.s004.pdf]

Underreporting

The underreporting in data collection is a fairly common problem in social sciences, public health, criminology, and microeconomics. It occurs when the counting of some event of interest is for some reason incomplete or there are errors in recording the outcomes. Examples are unemployment data, infectious or chronic disease data (e.g. HIV or diabetes), crimes with an aspect of shame (e.g. sexuality and domestic violence), error counts in a production processes or software engineering, and traffic accidents with minor damage [1]. An estimated prevalence of events based on the incomplete counts is likely to be smaller than the true proportion of events in the population. Several inference techniques based on binomial, beta-binomial, and regression models have been proposed for estimating the actual count values [2]. However, in all those techniques the reporting probability (underreporting rate) is assumed to be a constant parameter over time that is estimated based on the sample counts.

A very similar problem exists in preliminary or pilot clinical investigations, epidemiological surveys, and longitude studies where the objective is to estimate any possible clinical effect of a treatment or prevalence of a particular disease in a population of patients, but the prevalence of events can only be estimated by selecting a sample of patients from the population [3].

In all these situations, the prevalence of the events are estimated based on a random sample of events from the population, under the assumption that the sample set contains the same characteristics and distributions of the actual population, including those of the underreported and missing cases.

Furthermore, it is often required to perform a sample-size calculation based on confidence intervals in order to provide a precise estimate with a large margin of certainty and to make sure that the estimated proportion is close to the actual proportion with a high probability [3]. Confidence intervals for the proportions estimated based on samples from large populations and finite populations can be calculated by using the normal approximation to the binomial distribution as follows:

For large populations:

$$p \pm z_{1-\alpha/2}\sqrt{\frac{p(1-p)}{N}}$$

For finite populations:

$$p \pm z_{1-\alpha/2}\sqrt{\left[\frac{p(1-p)}{N}.\frac{N_{Population-N}}{N_{Population}}\right]}$$

where N is the size of sample, $p = \frac{r}{N}$ is the estimate of the proportion of events of interests in the sample and $N_{Population}$ is the size of population in case of finite populations [3].

In this study, we estimated the prevalence of adverse events by making sure that we have a significantly large enough number of samples to provide confident estimates. Our estimations are obtained under the assumption that the characteristics and distributions of the observed events are not significantly different from those in the actual population and would not significantly change after including the underreported cases. We are currently investigating the extension of the proposed inference techniques in [1][2] to estimate the actual number of adverse events with considering a variable reporting probability over time.

[1] Neubauer, G. and Friedl, H., "Modelling sample sizes of frequencies," *Proceedings of the 21st International Workshop on Statistical Modelling*, 3-7 July 2006, Galway, Ireland.

[2] Neubauer, G., Djuras G., Friedl H., "Models for underreporting: A Bernoulli sampling approach for reported counts," *Austrian Journal of Statistics*, Vol. 40 (2011), No. 1 & 2, 85–92

[3] Machin D, Campbell MJ, Tan S. Sample Size Tables for Clinical Studies. BMJ Books; 2008.
