# Supplementary material for: Adverse Events in Robotic Surgery: A Retrospective Study of 14 Years of FDA Data
Source: PLoS One. 2016 Apr 20;11(4):e0151470. doi: 10.1371/journal.pone.0151470 (PMC4838256; doi:10.1371/journal.pone.0151470)
Supplement: S1 Table — (PDF) [file pone.0151470.s005.pdf]

Robotic versus Non-robotic Surgical Methods

Previous studies on the effectiveness of robotic surgery and comparison of the outcomes to non-robotic minimally invasive methods fall into one of the following categories: 1) case-controlled studies that performed retrospective comparison of outcomes from robotic and non-robotic minimally invasive procedures done by specific surgical teams and institutes, 2) meta-analysis studies that systematically reviewed and combined the results from multiple studies. There are only a few small size randomized controlled trial studies that compared robotic and laparoscopic minimally invasive methods (e.g. one study compared robot-assisted laparoscopic radical prostatectomy (RALP) and retropubic radical prostatectomy (RRP) [1]). The case-controlled studies may suffer from selection bias in choosing the surgery methods for individual patients. Usually the less difficult cases are chosen early in the learning curve of a new procedure method, potentially leading to an unfair comparison of procedures. In addition, retrospective studies based on analysis of medical records may underreport actual rates of complications due to missing information in the records. Further, the unfavorable outcomes experienced during learning curve may not get published at all, leading to biased analysis of outcomes by meta-analysis studies [2].

S1 Table provides a summary of the previous studies across different surgical specialties [2]-[18]. For each specialty, we selected a sample of studies on the most common type of procedure performed in that specialty (e.g. hysterectomy in gynecology, prostatectomy in urology, and mitral valve repair in cardiology). We picked the case-controlled and meta-analysis studies that covered a large population of patients (n > 100 when possible) as well as randomized control trials, from the high impact journals published between 2007 and 2014. Most of the studies especially in gynecology and urology, for which the robots are extensively used, show better outcomes compared to other minimally invasive methods in terms of amount of blood loss during surgery, length of hospital stay, and mortality rates. However, the case-controlled studies report contradictory results on the mean operative time and complication rates in robotic versus laparoscopic hysterectomy and prostatectomy. This is because those factors are highly dependent on the expertise level of surgeon and the number of cases required to overcome the learning curve in robotic surgery [19]. Further, comparisons of outcomes for more complex procedures in cardiothoracic and head and neck surgery have rarely been done, and the existing studies often show that robotic approach is no more effective than non-robotic minimally invasive methods.

S1 Table. Related work on comparison of robotic vs. non-robotic minimally invasive surgical methods

Specialty	Study	Study Type	Surgical Procedure	Patients (n)	Mortality Rate (%)	Complication Rate (%)	Conversion/ Reoperation Rate (%)	Mean Operative Time(min)
Gynecology	Boggess et al. 2008 [3]	Cohort	Hysterectomy LAP	81	--	13.6	4.9	213.4
			ROB	103	--	5.8	2.9	191.2
	Gaia et al. 2010 [2]	Systematic Review	LAP	424	--	3.8	9.9	209
			ROB	396	--	2.0	4.9	219
	Wright et al. 2012 [4]	Database Analysis	LAP	2,464	0.2	9.8	--	--
			ROB	1,437	0.1	8.1	--	--
	Wright et al. 2013 [5]		LAP	4,971	0	5.3	0.1	--
			ROB	4,971	0	5.5	0.1	--
Urology	Rozet et al. 2007 [6]	Multiple Surgeons	Prostatectomy LRP	133	0	9.1	0	160
			RRP	133	0	19.4	3.0	166
	Berryhill et al. 2008 [7]	Meta Analysis	LRP	5,411	0	15.6	1.5	227
			RRP	5,472	1 death	6.6	0.5	164
	Porpiglia et al. 2013 [8]	Randomized Control Trial	LRP	60	--	11.6	--	138.1
			RRP	60	--	16.6	--	147.6
	Robertson et al. 2013 [9]	Systematic Review	LRP	4,952	0	0.76	0.3	238
			(*Predicted Prob.) RRP	6,768	0.2*	0.06	0.9*	225
General	Rawlings et al. 2007 [10]	Single Surgeon	Colectomy LAP	27	--	14.8	7.4	199.4
			ROB	30	--	20.0	6.6	225.2
	Müller-Stich et al. 2007 [11]	Randomized Controlled Trial	Fundoplication LAP	20	--	10.0	0	102
			ROB	20	--	15.0	0	88
	Breitenstein et al. 2008 [12]	Prospective Case-matched	Cholecystectomy LAP	50	--	2.0	0	50
			ROB	50	--	2.0	0	55
	Edelson et al. 2011 [13]	Retrospective Database Analysis	Gastric banding LAP	120	--	16.6	2.5	30.9
			ROB	287	--	17.1	3.1	91.5
Cardiothoracic	Mihaljevic et al. 2011 [14]	Single Institute	Mitral valve repair PST	270	0	9.9	2.6	277
			ANT	114	0	2.7	2.6	327
			ROB	261	0	11-12	9.1	387
	Swanson et al. 2014 [15]	National Database Analysis	Lobectomy VATS	295	--	18.98	--	253.8
			(*Major events) ROB	295	--	16.95*	--	269.4
			Wedge resection VATS	325	--	15.69	--	171.6
			(*Major events) ROB	325	--	21.58*	--	195.6
	Kent et al. 2014 [16]	National Database Analysis (Propensity-matched)	Lobectomy VATS	1,233	1.1	45.3	--	--
			ROB	411	0.2	43.8	--	--
Head & Neck	Lee et al. 2011 [17]	Retrospective Single Institute	Thyroidectomy END	96	--	10.4	0	142.7
			ROB	163	--	11.0	0	110.1
	Yoo et al. 2012 [18]	Retrospective Single Institute	END	165	--	11.5	0	145.2
			ROB	45	--	12.9	0	118.3

ROB: robotic, LAP: laparoscopy, LAPT: laparotomy LRP: laparoscopic radial prostatectomy, RRP: robot-assisted radial prostatectomy, PST: Partial sternotomy, ANT: mini-anterolateral thoracotomy, VATS: Video-assisted thoracic surgery

[1] Ficarra, Vincenzo, et al. "Retropubic, laparoscopic, and robot-assisted radical prostatectomy: a systematic review and cumulative analysis of comparative studies." *European urology* 55.5 (2009): 1037-1063.

[2] Gaia, Giorgia, et al. "Robotic-assisted hysterectomy for endometrial cancer compared with traditional laparoscopic and laparotomy approaches: a systematic review." *Obstetrics & Gynecology* 116.6 (2010): 1422-1431.

[3] Boggess, John F., et al. "A comparative study of 3 surgical methods for hysterectomy with staging for endometrial cancer: robotic assistance, laparoscopy, laparotomy." *American journal of obstetrics and gynecology* 199.4 (2008): 360-e1.

[4] Wright JD, Burke WM, Wilde ET, et al. Comparative effectiveness of robotic versus laparoscopic hysterectomy for endometrial cancer. *J Clin Oncol.* 2012; 30(8):783-91.

[5] Wright JD, Ananth CV, Lewin SN, et al. Robotically assisted vs laparoscopic hysterectomy among women with benign gynecologic disease. *JAMA.* 2013; 309(7): 689-98.

[6] Rozet, François, et al. "A direct comparison of robotic assisted versus pure laparoscopic radical prostatectomy: a single institution experience." *The Journal of urology* 178.2 (2007): 478-482.

[7] Berryhill, Roy, et al. "Robotic prostatectomy: a review of outcomes compared with laparoscopic and open approaches." *Urology* 72.1 (2008): 15-23.

[8] Porpiglia, Francesco, et al. "Randomized controlled trial comparing laparoscopic and robot-assisted radical prostatectomy." *European urology* 63.4 (2013): 606-614.

[9] Robertson, Clare, et al. "Relative effectiveness of robot‐assisted and standard laparoscopic prostatectomy as alternatives to open radical prostatectomy for treatment of localized prostate cancer: a systematic review and mixed treatment comparison meta‐analysis." *BJU international* 112.6 (2013): 798-812.

[10] Rawlings AL, Woodland JH, Vegunta RK, Crawford DL. Robotic versus laparoscopic colectomy. *Surg Endosc.* 2007;21(10):1701-8.

[11] Müller-Stich, B. P., et al. "Robot-assisted versus conventional laparoscopic fundoplication: short-term outcome of a pilot randomized controlled trial." *Surgical endoscopy* 21.10 (2007): 1800-1805.

[12] Breitenstein S, Nocito A, Puhan M, Held U, Weber M, Clavien PA. Robotic-assisted versus laparoscopic cholecystectomy: outcome and cost analyses of a case-matched control study. *Ann Surg.* 2008; 247(6):987-93.

[13] Edelson, Paula K., et al. "Robotic vs. conventional laparoscopic gastric banding: a comparison of 407 cases." *Surgical endoscopy* 25.5 (2011): 1402-1408.

[14] Mihaljevic, Tomislav, et al. "Robotic repair of posterior mitral valve prolapse versus conventional approaches: potential realized." *The Journal of thoracic and cardiovascular surgery* 141.1 (2011): 72-80.

[15] Swanson, Scott J., et al. "Comparing robot-assisted thoracic surgical lobectomy with conventional video-assisted thoracic surgical lobectomy and wedge resection: results from a multihospital database (Premier)." *The Journal of thoracic and cardiovascular surgery* 147.3 (2014): 929-937.

[16] Kent, Michael, et al. "Open, video-assisted thoracic surgery, and robotic lobectomy: review of a national database." *The Annals of thoracic surgery* 97.1 (2014): 236-244.

[17] Lee, J., et al. "Comparison of endoscopic and robotic thyroidectomy." *Annals of surgical oncology* 18.5 (2011): 1439-1446.

[18] Yoo, Ho, et al. "Comparison of surgical outcomes between endoscopic and robotic thyroidectomy." *Journal of surgical oncology* 105.7 (2012): 705-708.

[19] Lim, Sey Kiat, et al. "Current status of robot‐assisted laparoscopic radical prostatectomy: How does it compare with other surgical approaches?" *International Journal of Urology* 20.3 (2013): 271-284.
