# Supplementary material for: Adverse Events in Robotic Surgery: A Retrospective Study of 14 Years of FDA Data
Source: PLoS One. 2016 Apr 20;11(4):e0151470. doi: 10.1371/journal.pone.0151470 (PMC4838256; doi:10.1371/journal.pone.0151470)
Supplement: S2 Table — (PDF) [file pone.0151470.s006.pdf]

S2 Table. Summary of related work on failures of robotic surgical systems

Study (Year)	Surgery Types	Medical Institute	No. Cases	Total Number of Failures (Failure Rate) Types of Malfunctions	Converted	Rescheduled
Eichel [1] (2005)	Urologic	UC Irvine	200	Total = 5 (2.5%)<br>Software (4), Mechanical (1)	Laparoscopic (1) (0.5%)	N/A
Kozlowski [2] (2006)	Radical Prostatectomy (RLRP)	Virginia Mason Medical Center (VMMC)	130	Total = 6 (4.6%)<br>Setup joint (2), Software incompatible (1), Robotic arm malfunction (1), Power-off error (1), Monitor loss (1)	Laparoscopic (1) Open (1) (1.5%)	4 (3.1%)
Borden [3] (2007)	Laparoscopic Prostatectomy	Virginia Mason Medical Center (VMMC)	350	Total = 9 (2.6%)<br>Setup joint (2), Robotic arm (2), Camera (1), Power error (1), Console metal break (1), Software incompatible (1), Monitor loss (1)	Laparoscopic (1) Open (2) (0.9%)	6 (1.7%)
Zorn [4] (2007)	Radical Prostatectomy (RLRP)	University of Chicago Pritzker School of Medicine (2003–2006)	725	Total = 7 (0.96%)<br>(Recover. = 0.21%, Non-Recover. = .05%)<br>Power-up failure (1), Optical malfunction (3), Robotic arm (1), Camera (2)	Surgeon handicap (3)	4 (0.5%)
Fischer [5] (2008)	Radical Prostatectomy	Klinik Hirslanden, Switzerland	210	Total = 2 (1%)<br>Robotic arm (2)	Laparoscopic (2) (1.0%)	N/A
Lavery [6] (2008)	Radical Laparoscopic Prostatectomy (RALP)	11 Institutions 700 Surgeons	8,240	Total = 34 (0.4%)<br>Robotic arm (14), Optical system (14), Master malfunctions (4), Power supply/circuit (6), Unknown error (3)	Laparoscopic (2) Open (8) (0.1%)	24 (0.3%)
Ham[7]] (2009)	Radical Laparoscopic Prostatectomy	Yonsei University, Korea	1	Case report of Surgeon's console failure	Delayed 15 min	
Kim [8] (2009)	Urology, General, Obstetrics and Gynecology, Thoracic and Cardiac Otorhinolaryngology	Yonsei University College of Medicine, Korea (2005–2008)	1,797	Total = 43 (2.4%)<br>**Robot failures (24):** On/off failure (1), Console malfunction (5), Robotic arm (6), Optic system (2), System error (10)<br>**Instrument failures (19):** Shaft injuries (9), Wire cutting (2), Unnatural motion (2), Instrument tip (2), Limitation in motion (1)	Laparoscopic (2) Open (1) (0.2%)	N/A
Kaushik [9] (2010)	Robot-assisted Radical Prostatectomy (RARP)	Survey of 176 Surgeons from 4 Countries	N/A	Total failures = 260 (before or after surgery)<br>Robotic arm (38%), Camera (17.6%), Setup joint (13.8%), Power error (8.8%), Ocular monitor loss (8%), Instruments (7.6%), Console handpiece break (3%), Software (1.9%), Backup battery (0.3%), Instrument identification (0.3%)	Open (18.8%), Laparoscopic (15%), Another robot, with one fewer robotic arm (8.7%)	46 (57.5%)
Finan [10] (2010)	Gynecologic Oncology	Mitchell Cancer Institute, University of South Alabama (2006–2008)	137	Total = 11 (8%)<br>Robotic arm (2), Light or camera cord (2), Maylard bipolar (1), Power failure (1), Port problem (1), Others (3)	Delayed 25 min.	N/A
Mues [11] (2011)	Urology, Gynecology, Cardiothoracic, General surgery, Otolaryngology, Neurosurgery	Ohio State University Medical Center, James Cancer Hospital (2008–2009)	454	Tip cover failures = 12 (2.6%)<br>Significant patient complications (25%)	Repaired at the time of surgery	N/A
Agcaoglu [12] (2012)	General Surgery	Cleveland Clinic	223	Total = 10 (4.5%)<br>Robotic instrument (4), Optical system (3), Robotic arms (2), Robotic console (1)	Open surgery (6) (2.7%)	N/A
Chen [13] (2012)	Urological Surgery	Veterans General Hospital, Taiwan (2005–2011)	400	Total = 14 (3.5%)<br>Robotic arm/joint (11), Optical system (1), Power system/connector (1), Endoscopic instrument (1), Software incomp. (1)	Recoverable(10) Laparoscopy (3) (0.8%)	1
Buchs [14] (2014)	General Surgery	A Teaching Institution (2006-2012)	526	Total = 18 (3.4%)<br>Robotic instruments (9), Robotic arms (4), Surgical console (3), Optical system (2)	Laparoscopic (1) (0.2%)	N/A

[1] Eichel L, Ahlering TE, Clayman RV. Robotics in Urologic Surgery: Risks and Benefits. *AUA Update Series* 2005; 24(lesson 13): 106–111.

[2] Kozlowski PM, Porter CR, Corman JM. Mechanical failure rate of DaVinci robotic system: Implications for pre-op patient counseling [abstract 1159]. *J Urol* 2006; 175(suppl):s372–s373.

[3] Borden Jr, L. S., P. M. Kozlowski, C. R. Porter, and J. M. Corman. "Mechanical failure rate of da Vinci robotic system." *The Canadian journal of urology* 14, no. 2 (2007): 3499.

[4] Zorn, Kevin C., Ofer N. Gofrit, Marcelo A. Orvieto, Albert A. Mikhail, R. Matthew Galocy, Arieh L. Shalhav, and Gregory P. Zagaja. "Da Vinci robot error and failure rates: single institution experience on a single three-arm robot unit of more than 700 consecutive robot-assisted laparoscopic radical prostatectomies." *Journal of Endourology* 21, no. 11 (2007): 1341-1344.

[5] Fischer, Boris, Nadja Engel, Jean-Luc Fehr, and Hubert John. "Complications of robotic assisted radical prostatectomy." *World journal of urology* 26, no. 6 (2008): 595-602.

[6] Lavery, Hugh J., Rahul Thaly, David Albala, Thomas Ahlering, Arieh Shalhav, David Lee, Randy Fagin et al. "Robotic equipment malfunction during robotic prostatectomy: a multi-institutional study." *Journal of Endourology* 22, no. 9 (2008): 2165-2168.

[7] Ham, Won Sik, Sung Yul Park, Ho Song Yu, Young Deuk Choi, Sung Joon Hong, and Koon Ho Rha. "Malfunction of da Vinci robotic system—disassembled surgeon's console hand piece: Case report and review of the literature." *Urology* 73, no. 1 (2009): 209-e7.

[8] Kim, Won Tae, Won Sik Ham, Wooju Jeong, Hyun Jung Song, Koon Ho Rha, and Young Deuk Choi. "Failure and malfunction of da Vinci Surgical systems during various robotic surgeries: experience from six departments at a single institute." *Urology* 74, no. 6 (2009): 1234-1237.

[9] Kaushik, Dharam, Robin High, Curtis J. Clark, and Chad A. LaGrange. "Malfunction of the Da Vinci robotic system during robot-assisted laparoscopic prostatectomy: an international survey." *Journal of Endourology* 24, no. 4 (2010): 571-575.

[10] Finan, Michael A., and Rodney P. Rocconi. "Overcoming technical challenges with robotic surgery in gynecologic oncology." *Surgical Endoscopy* 24, no. 6 (2010): 1256-1260.

[11] Mues, Adam C., Geoffrey N. Box, and Ronney Abaza. "Robotic instrument insulation failure: initial report of a potential source of patient injury." *Urology* 77, no. 1 (2011): 104-107.

[12] Agcaoglu, Orhan, Shamil Aliyev, Halit Eren Taskin, Sricharan Chalikonda, Matthew Walsh, Meagan M. Costedio, Matthew Kroh et al. "Malfunction and failure of robotic systems during general surgical procedures." *Surgical Endoscopy* (2012): 1-4.

[13] Chen, Cheng-Che, Yen-Chuan Ou, Cheng-Kuang Yang, Kun-Yuan Chiu, Shian-Shiang Wang, Chung-Kuang Su, Hao-Chung Ho et al. "Malfunction of the da Vinci robotic system in urology." *International Journal of Urology* (2012).

[14] Buchs, Nicolas C., et al. "Reliability of robotic system during general surgical procedures in a university hospital." *The American Journal of Surgery* 207.1 (2014): 84-88.
