# Supplementary material for: Adverse Events in Robotic Surgery: A Retrospective Study of 14 Years of FDA Data
Source: PLoS One. 2016 Apr 20;11(4):e0151470. doi: 10.1371/journal.pone.0151470 (PMC4838256; doi:10.1371/journal.pone.0151470)
Supplement: S3 Table — (PDF) [file pone.0151470.s007.pdf]

S3 Table. Most frequent system error codes

System Error Code	Description	Type of Safe State that System Transits To	No. of Adverse Events
#20008	The angular position of one or more robotic joint's on the specified manipulator, as measured by the joint's primary control sensor (encoder) and the secondary sensor (potentiometer), were out of specified tolerance for agreement.	Recoverable	62
#23008			42
#20013			34
#23013			20
#21008			18
#21013			17
#23002			8
#20009			7
#22003			5
#212	A voltage-tracking fault reported by the digital signal processor (dsp) when the actual voltage to drive current through the motors deviates from the expected voltage by a specified amount.	Non-recoverable	31
#23	Hardware wheel "wdog" has tripped on one of the digital communication links in the system (due to an excessive number of retries on hardware message packets). This means that the system cannot reliably communicate over that digital link and therefore cannot continue normal operation.<br>Communication faults in the low-voltage differential signal carrying information about the patient side manipulator.<br>Communication faults between two system components.	N/A	28
#1	A power supply voltage was out of range.	Non-recoverable	19
#3	A redundant switch was missing its ground sense, or the contacts did not report as expected at startup.	N/A	15
#23017	A motor did not respond as expected, and the measured motion did not match the internal stimulation of the motor.	Recoverable	14
#2	A reference voltage was out of range.	N/A	14
#31030	One of the camera controller units in the doco has failed to power on after multiple attempts or has shut down after initially powering up.	N/A	14
#5	One or more fans are not moving as desired	N/A	10
#297	An electronic component was reporting an incorrect configuration.	Non-recoverable	9
#252	Master supervisory controller did not receive an expected message within a specified time.	Recoverable	8
#23020	One of the switches in a specific manipulator is showing inconsistent signals on its two switch leads.	Recoverable	7
#25589	During the power up self-test, the remote arm controller board (rac) brakes failed the brake voltage test.	Recoverable	6
#25588	A sympathetic error and occurs during the self-test upon system power-up when a loop response test fails.	Recoverable	6
#23007	On startup, one or more robotic joints on the manipulator did not make the prescribed test motion to within the specified tolerance.	Recoverable	6
#21003	The arm did not perform the commanded motions during startup within a specified tolerance.	N/A	5
#281	A processor did not complete a step during system startup within the allotted time.	Non-recoverable	5
#23034	After a specified amount of time, a valid event was not seen for one of the remote compute engine switches.	Recoverable	5
#45049	A communication timeout with the software running the da Vinci onsite application.	Recoverable	4
