# Supplementary material for: Adverse Events in Robotic Surgery: A Retrospective Study of 14 Years of FDA Data
Source: PLoS One. 2016 Apr 20;11(4):e0151470. doi: 10.1371/journal.pone.0151470 (PMC4838256; doi:10.1371/journal.pone.0151470)
Supplement: S4 Table — (PDF) [file pone.0151470.s008.pdf]

S4 Table. Example malfunctions and their patient impact during cardiothoracic procedures

MAUDE Report No. (Year)	Surgery Type	Event	Faulty Component	Patient Impact	Recovery Actions
932174 (2007)	Mitral valve repair	Possibly port placement; The robotic arms were never seen to collide, but this could have occurred, resulting in pressure on the retractor.	N/A	Left atrial disrupted, A 3 cm tear occurred in the hood of atrium medial to left atrial appendage, extending down towards the mitral annulus.	A patch was brought into place, trimmed, sewn using a suture, and tied. Sternotomy incision made for further repair.
1077464 (2008)	Beating heart double vessel CABG	Unexplained movement of the system arm with the endowrist stabilizer instrument attached to it. Feet at the distal end of the endowrist stabilizer tipped downward.	System arm	Damage to the myocardium of the patient's left ventricle.	Converted to open sternotomy and repaired.
1590517 (2010)	CABG	Micro bipolar forceps (mbf) instrument jumped forward. When master tool manipulator was moved, the instrument felt stuck and then moved.	Patient side manipulator (psm)	Patient's artery punctured.	Damaged section of artery was transected and the healthy portion was used to complete the bypass. Company replaced the psm component.
2494890 (2012)	CABG	Arcing from bipolar forceps instrument.	Forceps instrument	Small burn to diaphragm.	Connected ground pads and checked electrical surgical unit.
2816230 (2012)	N/A	Patient-side manipulator (psm) arm 2 jumped.	Patient-side manipulator (psm)	The forceps instrument on the psm lacerated the patient's mammary artery.	Converted to open surgery.
